# A pragmatic approach to identifying implementation barriers and facilitators for a novel pre-exposure prophylaxis (PrEP) delivery model at public facilities in urban Uganda

**DOI:** 10.1186/s43058-022-00254-w

**Published:** 2022-01-28

**Authors:** Dorothy Thomas, Andrew Mujugira, Katrina Ortblad, Sylvia Namanda, Joseph Kibuuka, Mai Nakitende, Florence Nambi, Lylianne Nakabugo, Caitlin Scoville, Timothy Muwonge, Renee Heffron

**Affiliations:** 1 Department of Global Health, University of Washington, Ninth and Jefferson Building, HMC 359927, 325 Ninth Avenue, Seattle, WA 98104-2499 USA; 2 Infectious Diseases Institute, Makerere University, Kampala, Uganda; 3 Public Health Science Division, Fred Hutchinson Cancer Research Center, 1100 Fairview Ave N, Seattle, WA 98109 USA; 4 Department of Epidemiology, University of Washington, Ninth and Jefferson Building, HMC 359927, 325 Ninth Avenue, Seattle, WA 98104-2499 USA

**Keywords:** CFIR, HIV, Uganda, PrEP, Implementation, LMIC, Barriers, Facilitators

## Abstract

**Background:**

Scalable HIV pre-exposure prophylaxis (PrEP) delivery models for resource-limited settings are critical for improving PrEP coverage and interrupting HIV transmission. This research uses technical assistance (TA) reports to evaluate implementation barriers and facilitators for a novel delivery model integrating PrEP and antiretroviral therapy (ART) delivery for HIV sero-different couples in public health facilities in Kampala, Uganda.

**Methods:**

We used data from the Partners PrEP Program (PPP)—a stepped-wedge cluster randomized trial that is launching PrEP delivery through an integrated model of oral PrEP and antiretroviral therapy (ART) delivery for HIV sero-different couples at public health facilities in Kampala and Wakiso, Uganda (NCT03586128). Technical assistance teams, comprised of PPP program staff, conducted monthly TA visits to implementing facilities where they identified and addressed implementation challenges in collaboration with health facility staff. Findings were recorded in TA reports, a standardized form structured using the Consolidated Framework for Implementation Research (CFIR). We used a conceptual content analysis approach to evaluate TA reports completed from January to December 2019 and identify implementation barriers and facilitators.

**Results:**

Among 39 reports from the 8 implementing facilities (~ 5 per facility), we identified 11 CFIR constructs. Key implementation facilitators included sensitizing and educating facility staff about PrEP (knowledge and beliefs about the innovation); establishing formal and informal feedback and accountability mechanisms (reflecting and evaluating); and empowering facility staff to address implementation challenges (self-efficacy). Key implementation barriers were related to ineffective recruitment and referral of sero-different couples to and from nearby facilities (cosmopolitanism) as well as stockouts of laboratory resources and testing supplies (available resources).

**Conclusions:**

This analysis featured a robust implementation science framework to assess the relationship between early implementation determinants and outcomes of this innovative PrEP delivery model. Further, we have provided important descriptions of early implementation barriers and facilitators that will inform scale-up efforts for PrEP delivery within and beyond Uganda. Future work will refine the analysis of pragmatic program data, qualitatively investigate the identified key themes, and explore strategies for addressing implementation barriers.

**Supplementary Information:**

The online version contains supplementary material available at 10.1186/s43058-022-00254-w.

Contributions to the literature

- To our knowledge, this study is the first to analyze technical assistance reports to determine early implementation barriers and facilitators using the Consolidated Framework for Implementation Research (CFIR).
- We have investigated the relationship between implementation determinants and outcomes of an innovative PrEP delivery model in a manner that facilitates comparison across studies.
- Findings from this research will inform the evaluation and effective implementation of PrEP delivery scale-up within and beyond Uganda.

## Background

Daily oral pre-exposure prophylaxis (PrEP) is a highly effective strategy for preventing HIV acquisition [1, 2] and efforts have now shifted to better understanding approaches for optimizing PrEP delivery and scale-up [3–5]. To date, pharmaceutical regulatory authorities in thirteen countries across sub-Saharan Africa, and over 100 countries, globally, have approved TDF-based medications for daily oral PrEP as HIV prevention [6, 7]. In 2016, Uganda began offering PrEP through the President’s Emergency Plan for AIDS Relief (PEPFAR) in select government health facilities and through demonstration projects and evaluations [6, 8]. Current public health delivery strategies prioritize PrEP access for certain key population groups including people who engage in transactional sex, men who have sex with men, people who are transgender and people who use drugs.

PrEP is a particularly applicable strategy for preventing the sexual transmission of HIV within the context of sero-different partnerships—in which one partner is living with HIV and the other partner is not. Sero-different couples are uniquely positioned to benefit from integrated antiretroviral therapy (ART) and PrEP strategies to prevent HIV transmission. In the Partners Demonstration Project, an open-label evaluation of integrated PrEP and ART use for HIV prevention among > 1000 heterosexual HIV sero-different couples in Kenya and Uganda, PrEP use was encouraged as a strategy to bridge the couple from a state of high acquisition risk to one with minimal risk due to sustained by ART use by the partner living with HIV. This strategy conferred a 96% reduction in HIV transmission for HIV-negative partners in the study cohort [9]. Conservatively, people living with HIV will achieve viral load suppression within six months of ART initiation. Thus, the time before ART initiation, and while ART use is becoming a sustained daily behavior, represents crucial periods for sero-different couples. These critical time periods underscore the importance of developing PrEP delivery models that are designed to address the specific circumstances of sero-different couples.

As PrEP programs continue to grow, pragmatic PrEP delivery models will be integral for maximizing coverage and suppressing HIV transmission. In order to facilitate the introduction and scale-up of PrEP delivery within and beyond Uganda, it is important to understand potential implementation barriers and facilitators. Implementation projects routinely collect programmatic data from supportive supervision and/or technical assistance visits in order to monitor the delivery of interventions such as PrEP for HIV prevention; however, these data are not typically subjected to formal analysis to inform real-time delivery. Using data from an implementation project assessing a novel model of PrEP delivery in urban Uganda, we analyzed monthly technical assistance (TA) reports using the Consolidated Framework for Implementation Research (CFIR) to identify early implementation barriers in facilitators. Technical assistance refers to guidance provided to entities such as governments, stakeholders or health facilities and that is intended to impart knowledge or skills in a given domain in order to realize progress [10]. We sought to identify contextualized findings about barriers and facilitators to launching PrEP delivery within existing ART programs. Our intention with this work is to expand understanding of barriers and facilitators to PrEP implementation success in order to facilitate the adoption and integration of evidence-based practices for optimized PrEP delivery models across similar regional and global settings. This pragmatic approach has implications beyond theoretical research projects and could be applied to inform the real-world scale-up of diverse health interventions including, but not limited to, PrEP delivery models.

## Methods

### Overview

For this evaluation, we used data from the Partners PrEP Program (PPP, #NCT03586128) which is a stepped-wedge cluster randomized trial that launched PrEP delivery to HIV-negative members of sero-different couples by integrating PrEP into existing ART programming in twelve public ART clinics in Kampala and Wakiso, Uganda. The main aim of the trial is to determine the impact of the integrated PrEP and ART delivery model on HIV viral suppression among partners living with HIV. Data on key outcomes, including PrEP initiation, PrEP refills, ART initiation, and HIV viral load, are abstracted from clinic records. In this trial, facilities were randomized (in groups of four) to one of three steps to begin intervention delivery: step 1 (first group to begin delivery), 2 (second group that begins delivery six months after step 1), or 3 (third group that begins delivery 12 months after step 1). When facilities moved from the control to intervention phase, providers from each facility were trained on PrEP delivery in a 2-day training, then PrEP medication was delivered to the facility and providers began offering PrEP to HIV-negative members of sero-different couples. At the start of the intervention phase, the PPP program staff (i.e., TA team) began routine visits and discussions with the facility staff about their experiences with PrEP delivery.

During their facility visits, the TA team collaborated with diverse health facility staff (e.g., nurses, clinical officers, counselors, pharmacists, PrEP champions and expert clients) to promote and support programmatic delivery by identifying and addressing implementation challenges. The TA team consisted of Ugandan research staff with established expertise conducting HIV prevention research, supporting PrEP providers and counseling research participants about PrEP [11]. Each member of the TA team possessed the language and cultural competency skills to effectively engage facility staff in their preferred language (i.e., either Luganda or English). TA visits were completed by the TA team over the course or two visits per month at each facility and lasted for roughly 2.5 h total (~ 1 h and 15 min for each of the two visits). Potential influences on the estimated duration of TA visits include client volume, data quality, if TAs were delivering specialized PrEP training to facility staff and if providers had conflicting tasks to tend to during the scheduled TA visits such that they were unable to meet with TAs or were otherwise unable to devote their attention. Nearly half of the TA visit was dedicated to reviewing PrEP-related registers and medical records; the remaining time was a balanced split between reviewing PrEP inventory and stock management practices, observing or interviewing facility staff to assess PrEP implementation practices, and reviewing laboratory sample collection practices as well as laboratory results. At the beginning of TA visits, facility staff shared status updates about pending action items and/or implementation challenges that they had experienced during the past month. At the close of these visits, the TA team collaborated with health facility staff to identify action items to address implementation challenges, determined a timeline for action item completion, and designated an action item “owner” who was responsible for its completion within the specified timeline. Throughout TA visits, the TA team worked closely with facility staff to build rapport and trust during in-person visits, in a WhatsApp group to share program updates and milestones, and during celebrations of facility successes.

### TA report data

We used the Consolidated Framework for Implementation Research (CFIR) to develop a standardized TA report intended to facilitate systematic data collection across facilities (see Appendix 1). We mapped the CFIR domains to the Partners PrEP Program implementation by reviewing and identifying the domains that were deemed most relevant to the intervention. Since TA reports were administered at health facilities with HIV providers, we excluded the intervention characteristics domain due to its emphasis on systems-level intervention barriers and facilitators. Thus, we developed the TA report to capture information on the CFIR domains of process, characteristics of individuals, inner setting, and outer setting. Guided by the selected CFIR domains, we structured the TA report to assess implementation performance through the following dimensions of the PPP implementation: (1) awareness and demand creation, (2) identification of PrEP users, (3) PrEP provision, (4) monitoring and follow-up of PrEP clients, (5) required resources for PrEP delivery, (6) monitoring and evaluation, and (7) implementation execution. Each dimension featured a set of associated questions that were intended to describe the extent to which PrEP delivery was successfully launched in implementing facilities. For example, the TA report featured questions such as “Are potential clients informed about PrEP during routine testing and risk counseling?” (awareness and demand creation), “Is partner status routinely assessed for individuals who get tested for HIV?” (identification of PrEP users), and “Are there bottlenecks or long waiting periods at certain stages/clinic areas?” (provision of PrEP). The TA team collaborated with facility staff to provide binary “Yes” or “No” responses to each question with the option to elaborate in a notes section, if needed. The TA team applied diverse approaches to collect TA report data, including direct observation of client care, review of medical records, review of ART and PrEP registers, and informal interviews with facility staff. Where relevant, the TA team triangulated data sources for each monthly TA report to identify as well as address areas of divergence. For example, to confirm the veracity of provider responses about PrEP registry data quality, TAs would review the PrEP registry to ensure alignment between provider responses and actual registry data quality. All TA reports were completed in Microsoft Word (Version 16.4).

### Data analysis

We defined the health facility as the unit of analysis and included data from PPP facilities that had at least six consecutive months of experience implementing the integrated PrEP and ART delivery model. We used the CFIR to guide data analysis [12, 13] and a conceptual content analysis approach to evaluate binary responses and open-ended notes within the TA report. The first step of our analysis was to develop one analysis memo per facility. This was done by transposing all monthly TA reports (i.e., up to 6 months of reports) for each facility into one facility-specific analysis memo. In order to code and evaluate the analysis memos, DT developed the study codebook by making minor modifications to the publicly available CFIR codebook template [14]. We used inductive and deductive reasoning to assess each analysis memo and identify codes to apply [15]. DT categorized responses and explicit notes outlined in analysis memos as implementation barriers or facilitators and assigned a corresponding CFIR domain (5 in total) and construct (39 in total). One advantage of coding binary data is relatively high agreement between researchers coding the same content. To reinforce the credibility of our analysis memo coding approach, authors DT, RH, and KO conducted member checks of the analysis memos and discussed discrepancies in categorization until agreement was achieved. We used Dedoose (Version 8.3) software for all analyses.

This analysis of the barriers and facilitators for integrating PrEP into the existing ART program in Uganda was collaboratively conducted by Ugandan and US American physicians and clinical researchers who possess hands-on experience with the realities of HIV treatment and prevention research in Uganda. After we completed coding analysis memos, a team of five analysts (DT, FN, MN, SN, JK) met weekly to collaboratively assign a valence score to each identified CFIR construct, indicating whether the construct manifested as a positive (+), negative (−), or neutral influence on the implementation [14].

After designating valence scores, we then assigned a strength score (options: 1 = weak or 2 = strong) to indicate the magnitude of each construct’s influence. We summarized valence and strength data across the analysis period and used a Microsoft Excel-based rating matrix to compare the valence and strength ratings for each construct across facilities. Finally, we assessed CFIR constructs for distinguishability to indicate whether the construct manifested similarly (non-distinguishing) or dissimilarly (distinguishing) across facilities.

## Results

Only eight (out of 12 total) facilities that had launched PrEP delivery during step 1 or 2 of the stepped-wedge trial were eligible for study inclusion. Half of the included facilities (*n* = 4) initiated PrEP delivery during step 1 of the implementation project and the other half (*n* = 4) during step 2, 6 months later. The global COVID-19 pandemic coincided with step 3 facilities starting PrEP delivery. Resultingly, data collection efforts were suspended as a pandemic safety response. Pandemic-related safety restrictions compromised PrEP delivery as well as TA report data collection for step 3 facilities for months three through six. Due to failure to meet the inclusion criteria of having 6 months of PrEP delivery data, step 3 facilities were excluded from the analysis.

**Fig. 1:**
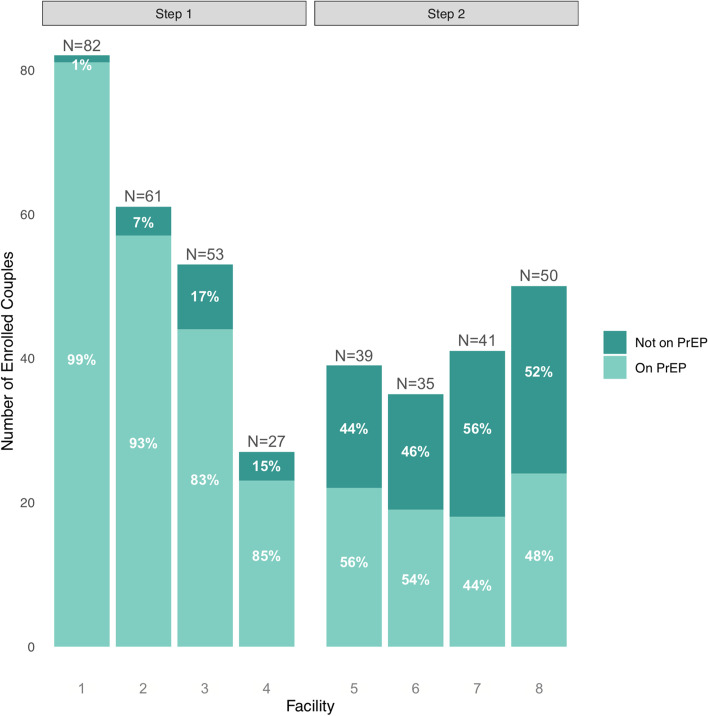
Number of clients enrolled and on PrEP from step 1 and 2 PPP facilities

Thus, we assessed the first six months of TA report data for the eight included facilities, representing the first 6 months of integrated PrEP and ART delivery to HIV sero-different couples in step 1 and 2 facilities. The eight implementing facilities featured a balanced mix of urban and peri-urban public health facilities with both Ministry of Health as well as NGO-based operating structures. Facilities served a median number of 720 ART clients per month (interquartile range [IQR] 590–1255 clients) and had a median number of 19 ART/PrEP providers (IQR: 11–20 providers). The median number of couples enrolled in PPP was 46 (IQR: 36–59 couples) per facility. From January to December 2019, 70% of HIV-negative members from eligible enrolled sero-different couples (90% in step 1 facilities and 50% in step 2 facilities) started PrEP within 30 days of study enrollment (Fig. 1).

### Outer setting domain

#### Cosmopolitanism (non-distinguishing)

This construct earned a mixed score indicating both positive and negative influences on implementation (Table 1). There were reports of unofficial client self-transfers to other ART clinics which made it difficult for providers to distinguish between clients who were lost to follow-up and those engaged in care elsewhere. Another example of the negative influence is that attempts to coordinate with nearby facilities to identify eligible sero-different couples were generally unsuccessful, despite persistent efforts. Referral challenges persisted despite frequent visits to nearby facilities to encourage referrals, creating referral agreements between facilities, offering incentives for referring providers, and availing resources (i.e., mobile phone airtime and transportation vouchers) to support external engagement with nearby facilities. An example of the positive influence is that facilities overwhelmingly reported effective and efficient sample transportation to external laboratories for testing.

**Table 1:** Partners PrEP Program CFIR construct valence and strength ratings

CFIR domain	CFIR construct	Strength and valence	Distinguishing
Outer setting	Cosmopolitanism	Mixed	
Inner setting	Networks and communication	+ 1	
	Available resources	+ 2	
	Access to knowledge and information	+ 1	
Characteristics of individuals	Knowledge and beliefs about the innovation	+ 2	
	Self-efficacy	+ 1	
	Other personal attributes	+ 1	
Process	Engaging champions	+ 1	X
	Engaging implementation participants	+ 2	
	Executing	+ 1	
	Reflecting and evaluating	+ 2	

### Inner setting domain

#### Networks and communication (non-distinguishing construct)

This construct earned a weak positive influence upon implementation success. An example of the positive influence is that there were consistent reports of effective linkages between the ART clinic and other departments in the facility. Facility leaders specifically identified informational talks with PPP program staff that counted as Continuing Medical Education (CME) credit, as an important opportunity for sensitizing facility staff in different departments about PrEP and integrating PrEP and ART services. The Maternal and Child Health department was also identified as an important aspect of the internal referral network for this implementation. The TA shared the following note:

“[Linkage between internal departments] is well done since all staff at these units are aware of services offered at the ART clinic, including PrEP.”

#### Available resources (non-distinguishing construct)

This construct earned a strong positive influence upon implementation. An example of the positive influence is that facility staff consistently reported having sufficient space and infrastructure to implement the intervention, secure areas to store client files, adequate HIV and hepatitis B testing kits, and consistent availability of Ministry of Health HIV reporting tools. While there were overwhelmingly positive reports of resource availability, some facilities also indicated a poor availability of creatinine reagents which are recommended but not required to be used to assess kidney function prior to ART and PrEP initiation for persons with comorbidities (e.g., diabetes, hypertension or cryptococcal meningitis).

#### Access to knowledge and information (non-distinguishing construct)

This construct earned a weak positive influence upon implementation. An example of the negative influence is that, due to administrative delays, facilities that first began delivering PrEP reported challenges with access to job aids and other PrEP communication materials. These challenges were counterbalanced by positive influences including the provision of checklists outlining strategies for incorporating the intervention into existing work tasks, offering trainings on PrEP to new facility staff, the provision of supportive supervision and informal training offered during TA visits. Other examples of the positive influence of this construct include the TA visits which helped to address staff questions and implementation challenges in real time, refresher trainings about calculating creatinine clearance, and the provision of a creatinine clearance calculator to support facility staff with this component of the implementation.

### Characteristics of individual domain

#### Knowledge and beliefs about the innovation (non-distinguishing construct)

This construct earned a strong positive influence upon implementation success. An example of the positive influence of this construct is that facilities reported that HIV providers and facility staff were knowledgeable about PrEP and PPP as a program. Facilities also reported that providers and staff in other departments had knowledge about PrEP; however, it was noted that in larger facilities, it was more challenging to coordinate efforts to ensure that all facility staff were knowledgeable about PrEP. Another challenge related to this construct was that some providers reported difficulty responding to client questions about concurrent condom and PrEP use, specifically, whether it is necessary to discontinue PrEP even when the partner living with HIV achieved viral suppression, and which common PrEP side effects to expect. A final example of this construct’s positive influence was the implementation of diverse strategies and activities to support PrEP and PPP knowledge including CME units and facility sensitization about PrEP and PPP as a program as well as formal and informal meetings with individual or multiple staff. The TA shared the following note:

“There has been a lot of sensitization about the PPP by both the TA and DAs [Data Abstractors] through CMEs [Continuing Medical Education units], one-on-one interactions, and all-staff meetings. This facility has no new staff who need training. After the meeting with all staff, everyone at the facility understands information about PrEP. The supervisors are not only now aware of PrEP but they are supportive of the PPP.”

#### Self-efficacy (non-distinguishing construct)

This construct earned a weak positive influence upon implementation success. An example of the positive influence of this construct is that facilities reported that providers were confident and comfortable when talking about PrEP to clients visiting the ART clinic. Another example of the positive influence of this construct is that facilities reported that providers were confident offering counseling and integrated PrEP and ART services for HIV sero-different couples.

#### Other personal attributes (non-distinguishing construct)

This construct earned a weak positive influence upon implementation success. An example of the positive influence of this construct is that facilities reported that health care workers were motivated to deliver PrEP to clients visiting the ART clinic. Another example of the positive influence of this construct is that facilities reported that providers possessed the skills and competence required for delivering PrEP. Despite facilities reporting that most providers had the motivation and competence to deliver PrEP, there were reports of individual staff members losing motivation to offer the intervention due to exclusion from training opportunities and being overburdened with other tasks. The TA shared the following notes:

“All those currently handling PrEP clients have the necessary skills and competencies.”

“Those involved in PrEP delivery have that self-motivation and are always willing to continue offering this service.”

### Process domain

#### Engaging champions (distinguishing construct)

This construct earned a strong positive influence upon implementation success in three facilities and was a distinguishing construct. The three facilities with strong positive scores for this construct indicated specific examples and instances in which facility staff went above and beyond to ensure implementation success by identifying and enrolling couples, proactively addressing implementation challenges, and demonstrating leadership and investment in intervention success. Two facilities with strong positive scores for this construct launched PrEP delivery in step 1 with an observed 99% and 93% PrEP uptake, respectively (i.e., facilities 1 and 2). The final facility with a strong positive score for this construct launched PrEP delivery in step 2 and had an observed 56% PrEP uptake (i.e., facility 5). This construct earned a weak positive influence on implementation success in five implementing facilities. Overall, all facilities indicated the positive influence of engaged PrEP champions.

#### Engaging implementation participants (non-distinguishing construct)

This construct earned a strong positive influence upon implementation success. An example of the positive influence of this construct is that facilities indicated the diverse ways in which they attracted and engaged HIV sero-different couples for intervention participation. For example, facilities reported displaying PrEP posters and sharing PrEP materials with clients. Additionally, they reported routinely assessing partner status for individuals seeking HIV testing and leveraging assisted partner notification services to recruit potential clients. Other examples of this construct included employing successful strategies for identifying and engaging sero-different couples (e.g., tracking and following up with clients who missed visits), leveraging clients’ social networks to obtain the contact information of friends and family (so providers could reach out to them for potential engagement), and using HIV self-testing and flexible HIV testing hours to accommodate clients’ schedules.

#### Executing (non-distinguishing construct)

This construct earned a weak positive influence upon implementation success in all facilities. One example of the positive influence of this construct is that facilities reported carrying out the PPP intervention as intended. Facility staff reported counseling HIV sero-different couples together, leveraging job aids and TA visits to promote implementation integrity and accountability as well as incorporating intervention activities alongside existing service offerings such as assisted partner services and referral of antenatal clinic clients. Implementation challenges related to this construct included inappropriate documentation of client information, national shortage of creatinine reagents which created challenges for assessing client creatinine levels, and inconsistent collection of viral load data.

#### Reflecting and evaluating (non-distinguishing construct)

This construct earned a strong positive influence upon implementation at all facilities. One example of the positive influence of this construct is that facilities indicated that there were feedback mechanisms in place to promote reflection and evaluation of intervention progress—both negative and positive. Facility staff also indicated that working with the TA team was useful for receiving feedback regarding the quality with which the intervention was delivered.

## Discussion

We leveraged TA report data to evaluate early implementation barriers and facilitators of an integrated PrEP and ART delivery model in Kampala, Uganda. To our knowledge, this is the first time that a rigorous implementation science evaluation framework has been used to analyze technical assistance data—a routine programmatic data source. Our findings indicate that it is feasible to implement integrated PrEP and ART services to members of HIV-sero-different couples in public health facilities in Uganda. We observed high levels of PrEP uptake, particularly in facilities with longer experience delivering PrEP. Key implementation facilitators emerged from the following CFIR constructs: available resources, knowledge and beliefs about the innovation, reflecting and evaluating, and engaging implementation participants. We identified the following facilitators to successful implementation of this integrated PrEP and ART delivery model: leveraging existing resources to engage HIV sero-different couples (e.g., assisted partner notification services, social network systems, flexible testing hours); integrating intervention activities alongside existing ART service offerings; acknowledging facility staff contributions to the intervention; promoting intervention fidelity and facility staff knowledge about the intervention by offering PrEP training during continuing medical education units, job aids, and supportive supervision during technical assistance visits; and establishing opportunities for facility staff to reflect on the intervention process and discuss implementation challenges and triumphs. In addition to implementation facilitators, we identified areas of improvement for launching PrEP delivery alongside existing ART programs. Our analysis revealed key implementation challenges including inconsistent collection of viral load data for partners living with HIV—an important metric for counseling HIV negative partners about PrEP continuation; provider difficulty responding to nuanced client questions regarding PrEP use; inadequate documentation of client information in facility files; and unofficial ART client self-transfers as well as ineffective recruitment of eligible individuals from external facilities.

Only one CFIR construct (engaging champions) manifested as distinguishing which suggests that our TA report evaluation revealed minimal diversity with respect to observed discrepancies in early implementation success across the eight health facilities included in this evaluation. The homogeneity in observed implementation barriers and facilitators may have been influenced by the relatively short implementation period that was evaluated. Additionally, the way in which the TA report is currently configured (e.g., consisting primarily of questions that solicit a binary response) may be insufficiently sensitive for assessing implementation heterogeneity over a brief implementation period. It is also important to note that this evaluation was conducted within the context of a stepped-wedge randomized control trial in which TA providers as well as health facility staff were well-resourced, highly invested, and motivated to ensure implementation success.

Further, we found that 10 out of 11 of the evaluated CFIR domains and constructs manifested in a positive manner (i.e., as implementation facilitators). This is an important contrast with existing evaluations of PrEP delivery interventions which have more readily identified barriers to implementation success. For example, an assessment across 5 sub-Saharan Africa countries found challenges of internal and external referrals due to overburdened staff to be an implementation barrier for PrEP delivery [16]. Similarly, an evaluation of PrEP delivery in Kenya reported challenges identifying and engaging potential PrEP clients due to ineffective referral networks [17]. A qualitative assessment of Kenyan providers’ experiences with PrEP implementation in Kisumu revealed challenges related to the documentation of client PrEP information, limited PrEP knowledge among providers and clients, multiple implementing partners operating in the same facilities but with different PrEP priorities and documentation practices, and providers citing competing responsibilities as important challenges or threats to PrEP delivery [18]. Further, several studies from resource-limited settings identified provider knowledge as an important influence for PrEP implementation [19–21]. One strength that we observed in this research was that the TA approach invited health facility staff into the implementation process as collaborators which subsequently empowered them to address intervention challenges as they arose. This emphasis on collaboration is well-aligned with findings from the PrEP scale up experience in Kenya in which a coordinated, collaborative PrEP scale up environment and high levels of internal ownership were identified as critical facilitators for implementation success [22].

Our findings provide a different, yet complimentary perspective to existing evaluations of PrEP delivery models in sub-Saharan Africa. In the present implementation, the identified challenges resonated with those from past research; however, in our study, these challenges dually manifested as opportunities for collaboration and problem solving. Although we identified PrEP delivery challenges that were similar to those in past research, our experience suggest that the identified implementation challenges were largely counterbalanced by facilitators that promoted knowledge and motivation for PrEP delivery among facility staff, empowered facility staff to identify and address challenges, and cultivated an enabling intervention environment that was made more efficient by leveraging existing strategies and service offerings for ART clients.

This analysis has important limitations for consideration. First, the TA report template largely featured binary “Yes” or “No” responses which offers limited richness with which to elaborate upon implementation experiences for this integrated PrEP and ART delivery model. Many implementation experiences may be too complex to be completely characterized with binary responses. Where available, we leveraged data in the “Notes section” of TA reports to better understand implementation experiences; however, not all TA reports offered this additional contextual information. Thus, there may be aspects of the identified barriers and facilitators in some facilities that are not reflective of the implementation environment in facilities for which there were more detailed qualitative information. Future research will explore opportunities to adapt this methodology for improved integration of more robust, nonbinary responses while simultaneously considering ease of reporting.

Second, ensuring that health facility staff are comfortable speaking openly about barriers to implementation can be challenging due, potentially, to anxieties of punitive action or fear of job loss if they report challenges. In an effort to address this limitation, our program TA team assured facility staff that their responses would be used for research purposes only to promote implementation success and, further, that their individual responses would be kept in confidence. Despite their knowledge of PrEP delivery and sincere efforts to encourage facility staff to provide reliable reports of their implementation experience, we acknowledge that research staff may introduce bias to TA data collection. With mounting interest in PrEP scale-up outside of a research context, there are imminent concerns regarding which health system staff will be best poised to provide and assess technical assistance—whether facility-level administrators, district level managers, or research staff. Finally, it is unclear to what extent these findings will be generalizable for PrEP delivery to populations beyond HIV negative members of sero-different couples. Future evaluations should refine and leverage more objective strategies for assessing determinants of success for PrEP delivery implementations in diverse pragmatic settings.

## Conclusion

These findings are timely and relevant since the national HIV prevention strategy in Uganda is inclusive of sero-different couples. With the forthcoming scale-up of PrEP delivery models within and beyond Uganda, studies like ours provide important evidence for identifying influences to PrEP implementation such that positive influences can be amplified, and negative influences can be repressed. This approach may serve as a catalyst for identifying and actualizing innovative PrEP delivery models in diverse settings. Although this research focuses on assessing an integrated PrEP and ART delivery model, our methodological approach may be more broadly applicable for diverse global health innovations, populations, and settings. In Uganda, findings from this work will inform best practices for successfully evaluating and implementing strategies that support the integration of PrEP delivery within existing HIV programs and services. Future work will explore the key themes identified in this analysis through in-depth qualitative interviews with staff from implementing facilities. Future research should evaluate the ways in which implementation success is influenced by the quality, quantity, and timing of TA provision. It will also be important to assess the cost of TA provision to gain insights regarding its return on investment. These findings will help to prevent HIV transmissions by supporting effective, broadly available PrEP delivery models in Uganda as well as in similar resource-limited settings.

## Supplementary Information

**Additional file 1.** StaRI checklist. We used the StaRI standards checklist to guide our description of our implementation science assessment and provide context regarding the primary intervention and related health outcomes.

**Additional file 2.** SRQR checklist. The SRQR checklist is reporting guideline for qualitative evaluations. We chose the SRQR checklist because it is well-aligned with the scope of work outlined in this proposal in which we wish to share new knowledge about assessing and addressing implementation challenges in order to improve healthcare.

**Additional file 3.** Appendix. Technical Assistance report template.

## Data Availability

Deidentified datasets generated and analyzed for the present study are available from the corresponding author upon reasonable request.

## Abbreviations

ART: Antiretroviral therapy
CFIR: Consolidated Framework for Implementation Research
CME: Continuing Medical Education
DA: Data abstractors
HIV: Human immunodeficiency virus
LMIC: Low- and middle-income countries
NGO: Non-governmental organization
PEPFAR: President’s Emergency Plan for AIDS Relief
PPP: Partners PrEP Program
PrEP: Pre-exposure prophylaxis
TA: Technical assistance/assistants
TDF: Tenofovir disoproxil fumarate
