# Supplementary material for: A pragmatic approach to identifying implementation barriers and facilitators for a novel pre-exposure prophylaxis (PrEP) delivery model at public facilities in urban Uganda
Source: Implement Sci Commun. 2022 Jan 28;3:7. doi: 10.1186/s43058-022-00254-w (PMC8795935; doi:10.1186/s43058-022-00254-w)
Supplement: Supplementary file 3 — Additional file 3. Appendix. Technical Assistance report template. [file 43058_2022_254_MOESM3_ESM.docx]

**Partners PrEP Program PrEP Phase Report**

| Facility name: |  |
| --- | --- |
| **Compiled by:** |  |
| **Other team members in attendance:** |  |
| **Visit dates:** |  |
| **Date of last visit:** |  |

| Pending action items from last report: | | Status and actions since last report: |
| --- | --- | --- |
| *1* |  |  |
| *2* |  |  |
| *3* |  |  |

**Site Visit(s):**

Use the TA visit prompts to document and elaborate on each of the following topics. Include the status and comments for each topic. Responses highlighted in grey require a comment. If you were unable to assess a topic, state “not able to assess” in the comments. In the support/training provided, write “n/a” if no support was provided or if no action points were agreed upon with facility staff during this visit.

| Topic | | yes | no | Comments | Support/training provided to clinic staff during visit |
| --- | --- | --- | --- | --- | --- |
|  |  | Note: grey responses require a comment | | |  |
| **Awareness & Demand Creation** | | | | | |
| *Potential users* | | | | | |
|  | Are PrEP posters on display? Where? |  |  |  |  |
|  | Are PrEP materials available or provided to clients? |  |  |  |  |
|  | Are potential clients informed about PrEP during routine testing & risk counseling? |  |  |  |  |
|  | Does the facility have: | | | | |
|  | Group health talks? |  |  |  |  |
|  | HIV prevention education? |  |  |  |  |
|  | Couples clubs? |  |  |  |  |
|  | Is PrEP included in these? (If “yes” comment which ones) |  |  |  |  |
|  | Are there other ways that the facility is recruiting PrEP clients? (e.g. outreaches, referrals from other facilities, individual referral) |  |  |  |  |
| *Providers* | | | | | |
|  | Do all HCWs in the ART clinic know about PrEP? |  |  |  |  |
|  | Do HCWs within the ART clinic routinely talk to clients about PrEP? |  |  |  |  |
|  | Do HCWs feel confident and comfortable talking about PrEP within the ART clinic? |  |  |  |  |
|  | Do HCWs and other staff in other departments know about PrEP (including supervisors, others)? |  |  |  |  |
|  | Are any HCWs PrEP champions? |  |  |  |  |
| **Identification of PrEP Users** | | | | | |
| *Couples and partner HIV counselling and testing* | | | | | |
|  | Is partner status routinely assessed for individuals who get tested for HIV? (i.e. do they ask about their partners status) |  |  |  |  |
|  | Do clients bring their partners to the facility for HCT? |  |  |  |  |
|  | Are there assisted partner notification (APN) services available? |  |  |  |  |
|  | Are there other ways that couples are being recruited for HCT? |  |  |  |  |
|  | Are providers confident providing couples HCT service, including couples counselling? |  |  |  |  |
| *Linkages and Referrals* | | | | | |
|  | Are there effective linkages between other departments at the facility and the ART clinic? |  |  |  |  |
|  | Are there effective referrals in place from other facilities for clients wanting to start PrEP? |  |  |  |  |
|  | If yes, are these referrals producing PrEP clients? |  |  |  |  |
|  | Are couples routinely recorded in the couples register at the ART clinic? |  |  |  |  |
| **Provision of PrEP** | | | | | |
| *Clinic Flow (follow a client through the different stages of PrEP delivery)* | | | | | |
|  | How long does it take the client to complete a PrEP visit? | n/a | |  |  |
|  | Are there bottlenecks or long waiting periods at certain stages/clinic areas? |  |  |  |  |
|  | Where/how can client flow be improved? | n/a | |  |  |
|  | Are PrEP services integrated with HIV+ client flow? |  |  |  |  |
|  | Are couples seen together for PrEP services? |  |  |  |  |
| *Counselling (observe counselling or consult with provider(s))* | | | | | |
|  | Are HCWs providing appropriate counselling (e.g. in line with training)? |  |  |  |  |
|  | Are there common questions or concerns among clients that are difficult to address? |  |  |  |  |
|  | How is adherence counselling and support provided? | n/a | |  |  |
| *Prescription and dispensing* | | | | | |
|  | Who is prescribing PrEP? | n/a | |  |  |
|  | Who is dispensing PrEP? | n/a | |  |  |
|  | Are there any differentiated care models for refills? |  |  |  |  |
| ***Monitoring & Follow-Up*** | | | | | |
| *HIV and Lab Testing (observation/review 5 random PrEP cards)* | | | | | |
|  | Do clinicians/HCWs request all required HIV and lab testing as per the national algorithm, including: | | | | |
|  | HIV? |  |  |  |  |
|  | Hepatitis B? |  |  |  |  |
|  | Creatinine? |  |  |  |  |
|  | Other? |  |  |  |  |
|  | How is creatinine clearance calculated at the facility and who is responsible? | n/a | |  |  |
|  | Are job aids posted in clinic rooms or otherwise available to HCWs? |  |  |  |  |
| *Case Management* | | | | | |
|  | What are the visit schedules being used for PrEP clients (e.g. asked to return after 2 weeks after PrEP initiation)? | n/a | |  |  |
|  | Are PrEP clients sent a visit reminder before their PrEP follow-up visit? |  |  |  |  |
|  | Are HIV- partners integrated into HIV+ client case management? |  |  |  |  |
|  | Do only certain providers see PrEP clients? |  |  |  |  |
|  | Are viral load tests for HIV+ partners of PrEP users routinely completed? |  |  |  |  |
|  | How are VL results reviewed and applied in case management for HIV- partners on PrEP? | n/a | |  |  |
|  | How are PrEP users monitored for side effects? (e.g. counselling, shorter time period for drug, phone number to reach clinician, etc.) | n/a | |  |  |
|  | Are PrEP clients seen at the ART clinic on specific days? |  |  |  |  |
| *Clients who miss PrEP visit(s)* | | | | | |
|  | Are PrEP clients tracked? How? | n/a | |  |  |
|  | Is the ART clinic tracking PrEP users who miss follow-up visits? |  |  |  |  |
|  | Are clients given reminder calls for missed appointments? |  |  |  |  |
|  | How does the clinic decide a client is lost-to-follow up? | n/a | |  |  |
| *Client Medical Records (review PrEP register and 5 random PrEP cards)* | | | | | |
|  | Where/how are PrEP records stored? | n/a | |  |  |
|  | Are providers adequately documenting services and information in the PrEP register? On the PrEP card? |  |  |  |  |
| **Required resources for PrEP delivery** | | | | | |
| *Workforce and infrastructure – across all areas (counsellors, clinicians, pharmacy, lab, etc.)* | | | | | |
|  | Are there adequate staff to deliver PrEP services? |  |  |  |  |
|  | Has there been staff turnover? |  |  |  |  |
|  | If yes, has staff turnover affected PrEP delivery? |  |  |  |  |
|  | Is there sufficient time to deliver PrEP services? |  |  |  |  |
|  | Do the HCWs have the skills and competencies necessary to delivery PrEP? |  |  |  |  |
|  | Are HCWs motivated to deliver PrEP services? |  |  |  |  |
|  | Is there adequate space and infrastructure for PrEP delivery? |  |  |  |  |
| *Commodities Management* | | | | | |
|  | Are drug accountability and tracking logs complete? |  |  |  |  |
|  | Bi-monthly provide a physical drug count: | n/a | | Number of bottles on-site: |  |
|  | Does the pharmacy have a process to request drug from IDIK? |  |  |  |  |
|  | Are HIV testing kits available? |  |  |  |  |
|  | Are HepB testing kits available? |  |  |  |  |
|  | Are there challenges within the commodities supply system (e.g. pharmacy/lab)? |  |  |  |  |
| *Laboratory systems* | | | | | |
|  | *Creatinine testing:* | | | | |
|  | Where is testing conducted? | n/a | |  |  |
|  | Does the testing facility have adequate reagents? |  |  |  |  |
|  | Are results provided in a timely manner from the testing facility? |  |  |  |  |
|  | Is HepB rapid testing correctly conducted and documented? |  |  |  |  |
|  | Is HIV testing correctly conducted and documented? |  |  |  |  |
|  | Are all lab results reviewed by a clinician in a timely manner? |  |  |  |  |
|  | Are lab samples effectively and efficiently transported to labs for testing? |  |  |  |  |
|  | Are lab samples consistently and accurately recorded? |  |  |  |  |
|  | Are there any other challenges related to the laboratory? |  |  |  |  |
| **Monitoring & Evaluation** | | | | | |
|  | Are there MOH M&E reporting tools available? |  |  |  |  |
|  | Are the MOH M&E tools fully completed? |  |  |  |  |
|  | Are there feedback mechanisms within the facility about how they are doing with PrEP delivery based on the M&E tools? |  |  |  |  |
| **Other** | | | | | |
|  | Are there any other items that are important to document? |  |  |  |  |

**Summary of training provided**

Use the below table to document formal training sessions held at the facility during the month reported:

| **Date of training** | **Type of training** | **Topics covered** | **Staff roles/departments in attendance** | **Trainer(s)** |
| --- | --- | --- | --- | --- |
|  |  |  |  |  |
|  |  |  |  |  |
|  |  |  |  |  |

**Source(s) of Information** (*check all that apply*)

□Direct observation of client care

□Medical records (e.g. PrEP facility card)

□Other (*specify*):

□Registers (*list specific registers that were useful*):

□Key personnel consultations (*list professional role (e.g.nurse*):

□Pharmacy □Peers □Doctor □Data

□Counselor □Couple’s focus person

□ Other, specify: __________

**Next Steps:**

Maintain an ongoing list of action items needed for each facility. At every visit, ensure you address all pending action item and document appropriately. Action items should be feasible and agreed upon by the facility staff and the PPP team.

| Issue needing follow-up | | Action plan | Person(s) responsible | Timeline | Status at time of report |
| --- | --- | --- | --- | --- | --- |
| 1 |  |  |  |  |  |
| 2 |  |  |  |  |  |
| 3 |  |  |  |  |  |
| 4 |  |  |  |  |  |
| 5 |  |  |  |  |  |
